# Enhanced strength–ductility synergy in ultrafine-grained eutectic high-entropy alloys by inheriting microstructural lamellae

**DOI:** 10.1038/s41467-019-08460-2

**Published:** 2019-01-30

**Authors:** Peijian Shi, Weili Ren, Tianxiang Zheng, Zhongming Ren, Xueling Hou, Jianchao Peng, Pengfei Hu, Yanfei Gao, Yunbo Zhong, Peter K. Liaw

**Affiliations:** 1 State Key Laboratory of Advanced Special Steel & Shanghai Key Laboratory of Advanced Ferrometallurgy & School of Materials Science and Engineering, Shanghai University, Shanghai, 200072 China; 2 Laboratory for Microstructures, Shanghai University, Shanghai, 200444 China; 3 Department of Materials Science and Engineering, The University of Tennessee, Knoxville, TN 37996 USA

## Abstract

Realizing improved strength–ductility synergy in eutectic alloys acting as in situ composite materials remains a challenge in conventional eutectic systems, which is why eutectic high-entropy alloys (EHEAs), a newly-emerging multi-principal-element eutectic category, may offer wider in situ composite possibilities. Here, we use an AlCoCrFeNi_2.1_ EHEA to engineer an ultrafine-grained duplex microstructure that deliberately inherits its composite lamellar nature by tailored thermo-mechanical processing to achieve property combinations which are not accessible to previously-reported reinforcement methodologies. The as-prepared samples exhibit hierarchically-structural heterogeneity due to phase decomposition, and the improved mechanical response during deformation is attributed to both a two-hierarchical constraint effect and a self-generated microcrack-arresting mechanism. This work provides a pathway for strengthening eutectic alloys and widens the design toolbox for high-performance materials based upon EHEAs.

## Abstract (web summary)

Producing in situ composite materials with superior strength and ductility has long been a challenge. Here, the authors use lamellar microstructure inherited from casting, rolling, and annealing to produce an ultrafine duplex eutectic high entropy alloy with outstanding properties.

## Introduction

Given the energy-efficient improvement and large safety factors, the attainment of high strength and high ductility in materials is arguably a vital requirement for most engineering applications. Unfortunately, these two properties are generally mutually exclusive, particularly in pure metals and alloys^1–6^. Eutectic alloys specifically exhibit good liquidity and castability, preventing common casting flaws, like internal shrinkage and compositional segregation, to downgrade mechanical properties^6^. They also possess a regularly arranged lamellar organization that can be viewed as a natural or in situ composite, which enables synergetic reinforcement to improve mechanical properties and sometimes bring unusual electrical, magnetic, and optical behaviors^7–11^. Eutectic materials have therefore been investigated in diverse application fields, but traditionally, only certain eutectic systems have been of major commercial importance^8–10^. Recently, eutectic high-entropy alloys (EHEAs)^11–13^, first proposed by Lu et al. in 2014^6^, were designed based on the above eutectic-alloy concept. Such alloys combine the advantages of both high-entropy alloys (HEAs)^14,15^ and conventional eutectic alloys, and usually show a fine dual-phase lamellar microstructure with scarce casting defects^6^. Yet, owing to the absence of mature design theories at present, there are only a few EHEA systems which possess an attractive tensile behavior^12,13^, which could benefit from being further optimized. Moreover, the safety and advancement of modern technologies not only strongly relies on current mechanical properties of these eutectic alloys, but also calls for better ones^7–10^.

To date, many conventional routes to optimize eutectic alloys, such as processing to create line defects, usually result in reduced ductility^6–8^. Previous studies showed that engineering an ultrafine-grained duplex microstructure by severe cold-rolling and annealing could dramatically strengthen eutectic alloys although degrading ductility^7^. Recently, Wu et al. developed a heterogeneous structure of bimodal grains in Ti, which possesses < 30 vol.% soft, coarse-grained lamellae embedded in a hard, ultrafine-grained lamella matrix^5,16^. The resulting materials exhibited great ductility, as a high density of lamella interfaces could induce the building of large strain gradients across them during deformation. Meanwhile, the full deformation constraint imposed by the hard lamella matrix enables the soft lamellae to be almost as strong as the hard matrix, thus making materials with high strength^16,17^. So a remarkable strength–ductility enhancement is expected if we could inherit the heterogeneous lamellar nature via an appropriate thermo-mechanical treatment, instead of just tailoring a dual-phase ultrafine-grained structure in eutectic alloys.

**Fig. 1:**
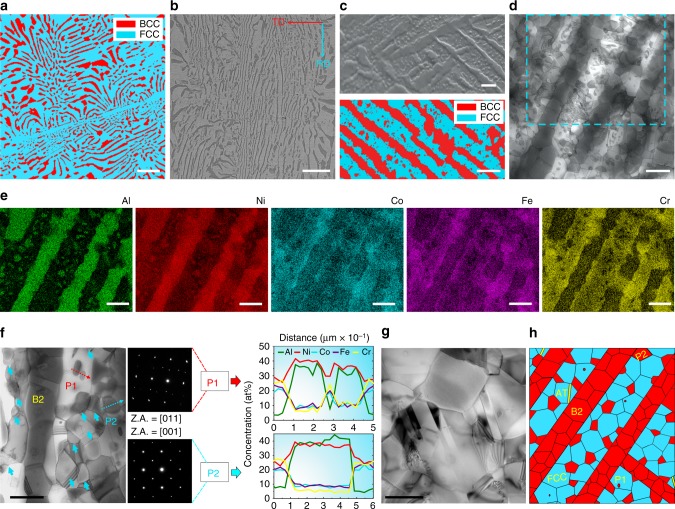
Microstructures of the as-cast EHEA and the hierarchical DPHL700. **a** Electron-backscatter-diffraction (EBSD) phase image of the as-cast EHEA. **b**, **c** Scanning-electron-microscope (SEM) image, high-magnification SEM image, and EBSD-phase image of the DPHL HEA. RD, rolling direction; TD, transverse direction. **d** Scanning TEM (STEM) image exhibiting a more detailed DPHL structure. **e** EDS maps of the identical region marked in **d** showing the distribution of Al, Ni, Co, Fe, and Cr. **f** Enlarged STEM image showing the distribution of P2 (the intergranular B2 phase, marked by blue arrows) and P1 (the intragranular B2 phase, marked by red arrow), and the corresponding SADPs and EDS composition profiles. **g** TEM image showing annealing twins. **h** Microstructural schematic sketch of the DPHL structure. AT, annealing twin. Scale bars, 20 µm in **a**, **b**, 2 µm in **c**, 1 µm in **d**–**f** and 500 nm in **g**

Inspired by the above idea, we architected a dual-phase heterogeneous lamella (DPHL) structure (Fig. 1) in a cold-rolled and annealed AlCoCrFeNi_2.1_ (at%) EHEA (detailed processing procedure shown in Methods). Compared to the aforementioned heterogeneous lamella structure, our current structure features the strength heterogeneity with soft/hard phases instead of bimodal grains^17^, and simultaneously exhibits a higher lamella density arising from the full lamella nature of eutectic alloys. Furthermore, owning to phase decomposition, there are substantial hard intergranular B2 (ordered-body-centered-cubic) precipitates in the soft FCC (face-centered-cubic) lamella matrix, thereby imparting an additional rigid deformation constraint to FCC grains^16,18^. Unexpectedly, due to the introduction of the DPHL structure, the as-fabricated EHEAs can activate microcrack-arresting mechanisms (the extrinsically ductilizing effect) to further extend the strain-hardening ability (the intrinsically ductilizing effect) for great ductility at the late stage of deformation. In this study, we prepared three EHEAs with the DPHL structure, and denoted them as DPHL660, DPHL700, and DPHL740 as per their different annealing temperatures, to study their mechanical behavior and deformation mechanisms.

## Results

### Microstructure characterization

Similar to the as-cast EHEA (Fig. 1a), the tailored DPHL HEA showed a typical lamella morphology. Different lamella domains exhibited varied inter-lamella spacings (1.5–5 μm) (Fig. 1b). These trends indicate the lamellar inheritance from the as-cast EHEA. There are also precipitated-out BCC (body-centered-cubic) phases of different sizes in the FCC lamellae (Fig. 1c). To better understand this microstructure, we conducted a detailed transmission electron microscopy (TEM) characterization coupled with energy-dispersive spectroscopy (EDS). Firstly, these lamellae consisted of recrystallized grains rather than simplex phase bands, and annealing twins were occasionally seen in FCC grains (Fig. 1d, g). Secondly, combining EDS maps and selected-area diffraction patterns (SADPs) suggested that the NiAl-rich lamellae (thickness of ~1 μm) were B2 grains, and the enriched Fe and Cr lamellae corresponded to FCC grains (Fig. 1e and Supplementary Fig. 1). The average diameters of FCC and B2 grains are roughly comparable (~0.71 μm). Here, we did not detect the Cr-enriched BCC nano-precipitate within the B2 lamellae in our DPHL HEA (Supplementary Fig. 2), although it is well documented that in the as-cast EHEA, the Cr-rich precipitates are densely dispersed inside the B2 lamellae^6,12^. Thirdly, as mentioned above, Fig. 1e, f also exhibited many BCC-phase precipitates in FCC lamellae. More specifically, they presented two types of NiAl-rich precipitates: the small and scarce P1 (intragranular B2 grains) of size 50–180 nm, and the large and primary P2 (intergranular B2 grains) with an average size of ~350 nm (Fig. 1f). Our results reveal a complex phase decomposition from the initial FCC lamellae^19^, which has now been detected in the EHEA category. This decomposition behavior has already been observed in some single-phase HEAs, such as the equiatomic CrMnFeCoNi HEA^19,20^, and originates from the limited entropic stabilization^19^. Before then, these alloys were widely accepted as a thermally stable single-phase solid solution, due to their ultrahigh mixing entropy^19–21^. Recently, Gwalani et al. linked the phase decomposition/precipitation incident with the competition between the thermodynamic driving force and activation barrier for the second-phase nucleation, synchronously coupled with the kinetics factor^22^. They suggested that the heavy cold-rolling provided high-energy interfaces, such as slip/twin bands and dislocations cell walls, which could act as heterogeneous nucleation sites for precipitates during annealing, consequently enabling a reduced heterogeneous nucleation barrier for the intergranular B2 and even σ phases^22^. Accordingly, as schematically illustrated in Fig. 1h, the developed DPHL structure shows a two-hierarchical heterogeneity^2,16–18^, which is composed of the submicron-grade FCC/B2 grains within the FCC lamellae and the micron-grade alternate FCC/B2 lamellae. Such structural characteristics were also seen in the other two DPHL HEAs (Supplementary Fig. 1).

### Tensile properties

**Fig. 2:**
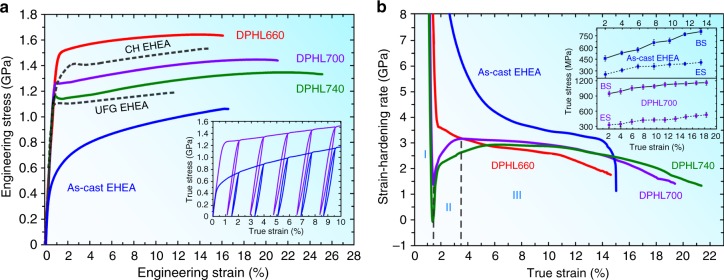
Mechanical responses of the three DPHL HEAs at room temperature. **a** Tensile properties. UFG EHEA and CH EHEA refer to the ultrafine-grained EHEA^7^, and the complex and hierarchical EHEA^23^, respectively. The inset is the loading–unloading–reloading behavior of the DPHL700 and the as-cast EHEA (whole curves exhibited in Supplementary Fig. 3). **b** Strain-hardening response. As a representative, the multistage stress–strain relationships are marked as I–III in DPHL700. The inset shows the back-stress (BS) and effective-stress (ES) evolution with the plastic strain for the as-cast EHEA and DPHL700. Error bars in the inset indicate standard deviations for five tests

Figure 2a displays the mechanical behavior of three DPHL HEAs (detailed properties listed in Supplementary Table 1). To emphasize the markedly improved properties after engineering the DPHL structure, the curves of the ultrafine-grained EHEA^7^ and the as-cast EHEA are also shown. Deploying an ultrafine-grained duplex microstructure makes the EHEA twice stronger in yield strength than its as-cast sample, but comes at loss of ductility^7^. In contrast, our DPHL700 and DPHL740 with the inherited lamellar geometry exhibit a simultaneous strength–ductility enhancement, and even higher strengths over that of the ultrafine-grained EHEA. Recently, Bhattacharjee et al. processed a complex and hierarchical microstructure in the same AlCoCrFeNi_2.1_ EHEAs by heavy cryo-rolling and annealing^23^, which shows a better strength–ductility balance (yield strength of ~1.437 GPa and ductility of ~14%) than the ultrafine-grained EHEA^7^ and a comparable property combination to that of our DPHL660 (Fig. 2a). However, it is noted that the tensile data in ref. ^23^ is not consistent with its stress–strain curve, and the real data ought to be yield strength of ~1.15 GPa and ductility of ~14% from the curve. This difference might be due to the abnormal tensile behavior after yielding (Fig. 2a). The dimensions of the tensile specimens in ref. ^23^ show that the sample is small, and the gage length, width, and thickness are 2 mm, 1 mm, and 300 μm, respectively, while only one annealing condition, 800 °C for 1 h, is applied. Thus, it is expected that uncertainty in the measurement of the properties could be larger due to possible drawbacks in small tensile specimens. In contrast, our slab cast by suction in the present research has a thickness 6 mm, which is double than that of ref. ^23^. We use three annealing conditions of 660, 700, and 740 °C for 1 h, and the dimensions of tensile specimens are 15 mm in gage length, 3.2 mm in width, and 600 μm in thickness, which are typically consistent with the frequently reported dimensions in the literature^16,18^. All tensile tests were conducted, using a 12-mm extensometer to monitor the strain. All these features make the present tensile data more persuasive in comparison to ref. ^23^. That’s also why the above three data points in the banana curves are aligned at the same level of excellence, as revealed in our next discussion. Certainly, there are a few other routes (for instance, preparing nano-lamellar or near EHEAs) to strengthen the present EHEAs, but these strategies have not led to significant improvements in tensile properties^12,23^. To the best of our knowledge, the strength–ductility combination achieved in our work is not accessible to previously reported reinforcement methodologies.

Figure 2b gives the strain-hardening rate (*Θ*) versus true strain curves. Interestingly, *Θ* first drops quickly in region I, even to below zero in the curve of DPHL740, then is followed by an up-turn to reach its maximum in region II, which is often observed in heterogeneous structures recently^16,18,24^. Wu et al. reported that such transient behavior is due to the lack of mobile dislocations^16^, which can not effectively accommodate the imposed constant strain rate at the onset of plastic deformation in region I. Upon yielding, dislocation multiplication and tangle is responsible for the rapid *Θ* increase^16^ in region II. Due to a few regions with a high dislocation density before the tensile deformation in our DPHL660 (Supplementary Fig. 1a), the resulting strain-hardening curve shows an obviously weakened transient behavior in Fig. 2b. In addition, the as-built DPHL700 shows a striking ability of sustaining high *Θ* over a wide strain region III, which is a prerequisite for attractive tensile ductility^1–3^. To better understand the origin of the observed high yield strength and strain hardening, we conducted loading–unloading–reloading (LUR) testing. The inset of Fig. 2a shows two LUR curves of the DPHL700 as a representative of DPHL-HEA samples and the as-cast EHEA for comparison. Both of them exhibit a hysteresis loop, which reveals the existence of Bauschinger effect^16^. Here, as reported in ref. ^16^, we divided the flow stress into the back-stress associated with a long-range stress on mobile dislocations and the effective stress required locally for the dislocation movement. Additionally, we estimated the contributions of the above two kinds of stresses to the flow stress from the LUR curves^24^ (inset in Fig. 2b). Overall, these mechanical features indicate that we could optimize eutectic-alloy properties by inheriting its composite lamellar nature in the ultrafine-grained EHEAs.

## Discussion

After achieving the desirable properties in the AlCoCrFeNi_2.1_ EHEAs, it naturally follows to analyze the strengthening mechanisms lying behind the enhanced strength–ductility synergy. Here, only upon a clear comprehension of the deformation processes of the hierarchical DPHL HEAs can we reasonably account for the observed mechanical response.

**Fig. 3:**
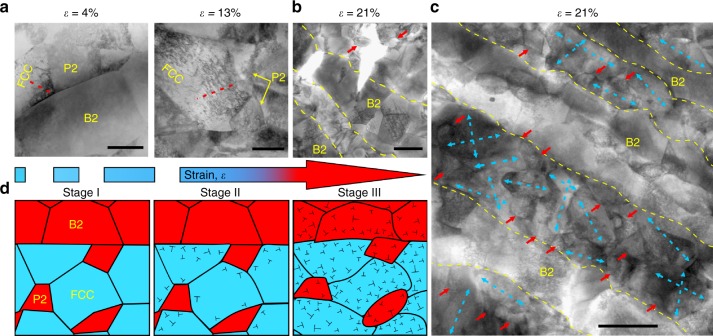
Deformation micro-mechanisms in the hierarchical DPHL HEA with the increasing tensile strain. **a** STEM images showing the dislocation-substructure evolution. The early stage of deformation (*ɛ* = 4%) leads to more obvious dislocations in soft FCC grains than hard P2 (the intergranular B2 grains) and B2 grains near phase interfaces. At medium strains (*ɛ* = 13%), there exhibit significantly-increased dislocations in FCC grains and P2. The piling-up of GNDs is marked by dashed red lines in FCC grains. **b**, **c** STEM images of the microstructure stretched to fracture (*ɛ* = 21%). The dual-phase lamellae and P2 (indicated by yellow dashed lines and red arrows, respectively) show apparent dislocations. **b** Microcrack propagation stays confined/blunted by neighboring lamellae. **c** The dashed blue arrows point out different deformation directions, and even some FCC grains deform along two directions. **d** Schematic illustration of the dislocation evolution during deformation. Stage I: elastic deformation; Stage II: elastic-plastic deformation; Stage III: plastic deformation. ⊥, dislocation. Note that stage I in **d** is not the schematic illustration of *ɛ* = 4% in **a**. Scale bars, 200 nm in **a**, **b** and 1 µm in **c**

First, we systematically discuss the high yield strength (Fig. 2a). During tensile deformation, after the soft FCC and hard B2 phases co-deformed elastically, the soft FCC lamella matrix is more susceptible to starting plastic deformation (Fig. 3a, d). Nevertheless, the soft FCC matrix cannot plastically deform freely, owning to the constraint by the still elastic B2 lamellae. Considering the strain continuity, this implies the existence of plastic-strain gradients in the soft lamella matrix near lamella interfaces^3,16^. Accommodation of such strain gradients needs the storage of geometrically necessary dislocations (GNDs). Consequently, this process produces a long-range back-stress, making dislocations difficult to move in FCC grains until B2 grains start to yield deformation^5^. Here it should be mentioned that the coarse-grained FCC matrix is synchronously surrounded by the intergranular B2 phase in FCC lamellae (Fig. 3a and Supplementary Fig. 4). As per the analysis above, the same back-stress is also produced, yet in another size^18^. Ultimately, under such two-hierarchical constraints, the FCC grains appear much stronger than when they are not constrained, producing so-called synergetic strengthening and significantly elevating the material yield strength^17^. Meanwhile, as shown in the inset of Fig. 2b, the back-stress (~950 MPa) of the closest to yield point is ~3 times higher than the effective stress in DPHL700, which quantitatively explains that the larger back-stress is primarily responsible for the observed high yield strength^24^. The high back-stress can therefore be regarded as a long-range internal stress connected with a local strain process, which enables the long-range interaction with mobile dislocations^16–18^. Correspondingly, the low effective stress is therefore the stress required locally for a dislocation to move, which is related to the short-range interaction in a similar way to friction stress and forest hardening^16–18^.

Second, we focus on the extra strain-hardening capacity prevailing in stage III (Fig. 2b). Beyond the yield point, both the soft and hard lamellae will deform plastically (Fig. 3a, d). But because the soft FCC grains deform easily, the FCC lamella matrix bear more plastic strains than hard B2 lamellae. Further strain gradients therefore appear^17^. However, the strain gradients in this case are built up not only in the soft but also hard lamellae near lamella boundaries. These strain gradients will become larger with ongoing deformation and thereby require more GNDs, leading to high back-stress hardening^1,17^. According to this theory, the same strain gradients and thus back-stress hardening will also appear in FCC lamellae due to the intergranular B2 phase^5^. Ultimately, they contribute to the observed high strain hardening together. This implies that an increased hard/brittle B2-phase content may still lead to the DPHL HEAs sustaining large or higher ductility^12,25^, in contrast to the as-cast EHEA.

Similarly, the excellent ductility for the above complex and hierarchical EHEA mainly stems from the strong back-stress hardening effect^23^. As reported by Bhattacharjee et al., a developed hierarchical microstructure is composed of two parts: the fine dual-phase lamellar region and the coarse dual-phase non-lamellar region^23^. Such a hierarchical architecture can provide substantial domain boundaries separating areas of diverse hardness, consequently being particularly favorable to benefiting from back-stress hardening and thus great ductility^16,18,23^. In the hierarchical structure, there are coarse non-lamellar regions featuring a mixture of soft FCC and hard B2 phases. Although these regions allow significant strain partitioning to induce back-stress strengthening, the soft FCC phase also allows the material to yield at low stress, owning to the absence of the lamellar constraint effect^17^. That is why a previous hierarchical structure^23^ shows a limited improvement in the yield strength compared to the corresponding ultrafine-grained EHEA^7^. Recently, Yang et al. reported work in a single-phase medium-entropy alloy^26^, where they purposely architectured a three-level heterogeneous grain structure with grain sizes spanning the nanometer-to-micrometer range via partial recrystallization annealing following conventional cold-rolling, achieving large uniform tensile strain (~22%) after yielding even at the gigapascal stress. They attributed these improved properties to high back-stress strengthening caused by structurally inhomogeneous deformation characteristics. On the one hand, the partially recrystallized starting structure with heterogeneous grain sizes supports the inhomogeneous plastic strain. On the other hand, the heterogeneous structure becomes even more heterogeneous during tensile straining as more twins, faults, and nano-grains with high-angle grain boundaries are generated dynamically owing to the low stacking fault energy, as well as the dislocations, leading to increased inhomogeneous plastic deformation. This implies a dynamically reinforced heterogeneous grain structure inducing strong back-stress hardening. In our work, the DPHL structure maintains its heterogeneous configuration during the entire plastic deformation which is only assisted by dislocations, without additional deformation mechanisms observed (Fig. 3c). Furthermore, compared to the above heterogeneous grain structure, our DPHL structure exhibits high hetero-interface density^16,17^ due to its composite lamellar nature, leading to an enhanced back-stress hardening potential^16,17^ regardless of additional mechanisms introduced during tensile deformation.

To further verify that the observed mechanical response is led by the inherited lamellar geometry in the ultrafine-grained EHEAs, we analyze the microstructure of DPHL700 stretched to fracture. As shown in Fig. 3c, the originally equiaxed grains were subjected to a large amount of inhomogeneous plastic deformation, and the initially smooth lamella interfaces became ragged and convoluted. Unexpectedly, FCC grains deformed along different directions, not a single tensile direction (Fig. 3c and Supplementary Fig. 4). These phenomena hint that the two-hierarchical constraint deformation effectively built up, and the dynamic hardening effect violently happened^5^ (Supplementary Fig. 4). Besides massive dislocations in FCC grains, there are also pronounced dislocations in both B2 lamellae and intergranular B2 grains (Fig. 3c), which shows that B2 grains can also plastically deform and sustain strain hardening in tension^18^. This provides direct evidence that forest dislocations have mediated dislocation hardening and contributed to the observed high strain-hardening capability^16^. In contrast, in the as-cast EHEA, many dislocations are generated/blocked in the soft FCC phase near phase boundaries, while no obvious dislocations are observed in the hard B2 phase^12^. This phenomenon is reflected by the fast-growing back-stress and little-varied effective stress in this investigation^27^ (inset in Fig. 2b). Eventually, this process causes strain localization (Supplementary Fig. 5) rather than kinematic strain partitioning, degrading the EHEA’s properties^5,27^. We, therefore, conclude that the ultrafine-grained EHEAs with the inherited lamellar architecture promote the improved properties. A further corroboration to the importance of the inherited lamellar nature is given when we modify our specimen by increasing the annealing temperature (900 °C) to induce a less ideal, partially degraded lamellar structure (Supplementary Fig. 1c, d), which leads to inferior tensile properties (Supplementary Fig. 6). Such an observation also lends support to the aforementioned assertions about deformation processes and strengthening mechanisms.

**Fig. 4:**
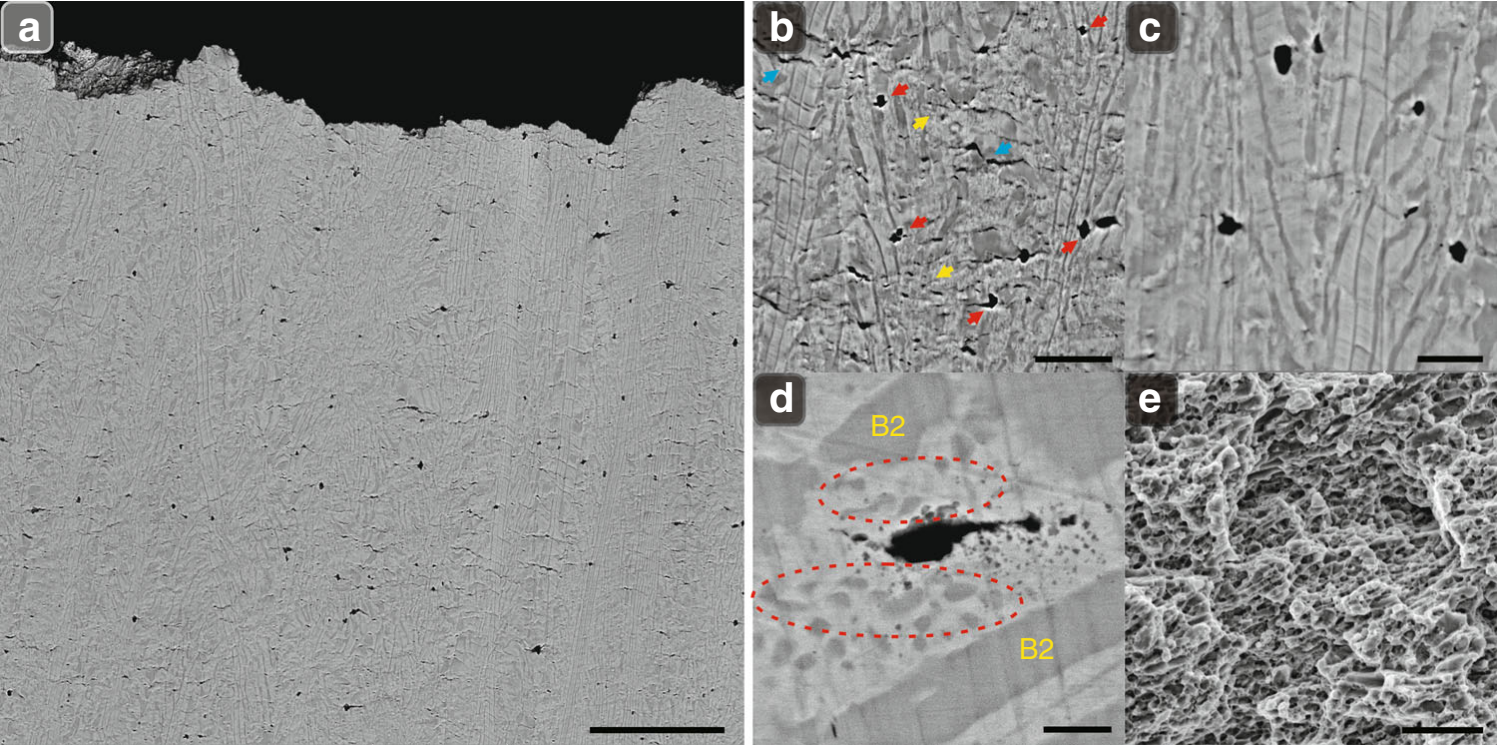
Typical SEM images of damage-evolution mechanisms for the hierarchical DPHL HEA at the late stage of deformation. **a** Microcrack distribution near the fractured end. **b** Multiple microcracks mainly including circle-like, tortuous, and even submicron cracks (indicated by red, blue, and yellow arrows, respectively). **c** At strains of 17–20%, the circle-like microcracks with blunted crack-tips, whose propagation remains confined by adjacent ductile components. **d** High-magnification SEM image indicating that these circle-like cracks are initially nucleated predominantly in FCC lamellae and caused by the limited deformation ability of P2 (the intergranular B2 phase, marked by dashed red circles), which are revealed at the strain of ~17%. **e** Fractography of the fracture surface with massive dimples, providing an indirect explanation for the enhanced ductility. Scale bars, 50 µm in **a**, 10 µm in **b**, 5 µm in **c**, 1 µm in **d**, and 5 µm in **e**

Furthermore, we analyze the damage-evolution mechanisms of DPHL700 at the late stage of deformation. There are extensive uniformly distributed microcracks near the fractured end, instead of large (secondary) cracks usually seen in most cases (Fig. 4a). These microcracks exhibit varied and complex morphologies, and include circle-like, tortuous, and even submicron cracks. Here, the circle-like cracks appear to be the primary damage incident, with additional long and tortuous cracks (Fig. 4a, b). During deformation, such multiple microcracks can effectively weaken the high localized stress^28^. However, in our case, the formation of large cracks by the coalescence of small microcracks was found to be inhibited, even when some of the microcracks are separated by only a single lamella (Fig. 3b and Fig. 4b). To elucidate this phenomenon, further microstructure observations found that after the heavy-rolling deformation (84–86%), the resultant samples possess massive special lamellae (~82–87 vol.%) with a directionally aligned arrangement along the rolling direction^29^ (Supplementary Fig. 7). It should be noted that the aligned arrangement here does not refer to the orientation behavior as in crystallography. With their special lamellar architecture, the DPHL materials are akin to artificially fabricated laminated composites^30^. The ductile components such as the FCC matrix, and even the hard B2 lamellae in the current samples, will therefore work well as very efficient crack arresters to delay crack propagation and coalescence during deformation:^30^ this is experimentally supported by the existence of restrained circle-like microcracks with blunted crack-tips (Fig. 4c, d). Eventually, these incidents delay the onset of global damage, enabling the further intrinsic kinematic hardening and inducing additional ductility (Fig. 4e). Such damage-evolution process does not take place in the as-cast EHEA even though they have a similar dual-phase lamellar structure, as these cracks with a simplex shape are primarily nucleated at the phase boundaries of the as-cast EHEA. Once initiated, they rapidly start to propagate and coalesce into long and large cracks along the lamellar interfaces due to the internal-stress accumulation and strain localization in FCC lamellae near phase boundaries^28^ (Supplementary Fig. 5).

**Fig. 5:**
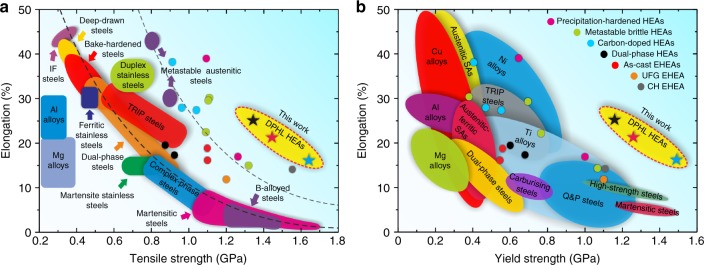
Tensile properties of the hierarchical DPHL HEAs in comparison with the traditional metallic materials and the previously-reported hardened HEAs. **a**, **b** A general summary of the fundamental tensile properties at ambient temperature. Note that the reported HEAs, including the as-cast EHEA^12,13^, ultrafine-grained (UFG) EHEA^7^, complex and hierarchical (CH) EHEA^23^, dual-phase HEAs^33^, metastable brittle HEAs^4^, precipitation-hardened HEAs^32^, and carbon-doped HEAs^31^, are the products of several of the most effective strengthening mechanisms among HEAs in general, but they only show the better tensile strength–ductility synergy, in comparison to traditional alloys. There, of course, are a few advanced HEAs not shown for comparison, due to their low yield strength (for example, < 400 MPa)^35^ and poor room-temperature properties

Figure 5 shows a comparison of tensile properties of the hierarchical DPHL HEAs with various advanced steels, traditional alloys, and other reported HEAs with superior mechanical properties^4,7,12,13,15,23,31–33^. In Fig. 5a, both the current DPHL HEAs and previously reported HEAs are separated from the general trend for conventional metallic materials, suggesting a favorable tensile strength–ductility combination. However, in the yield strength-elongation map (Fig. 5b), only our DPHL HEAs stand out from the trend. This reveals a common phenomenon/problem that it is currently possible (though difficult) to achieve great tensile strength–ductility balance in HEAs, but very challenging to simultaneously possess sufficiently high yield strength. Recently, Liang et al. prepared ultrastrong precipitation-hardening high-entropy alloys (PH-HEAs)^34^. Different from the common PH-HEA design idea, they explored a non-equiatomic alloy as a prototype specimen, and then utilized spinodal decomposition to create a low-misfit coherent nanostructure. This enhanced the ability to achieve higher contents of nano-precipitates, and also helped obtain a near-equiatomic, high-entropy FCC matrix enabling ultrahigh yield strength (~1570–1810 MPa) while retaining good ductility (~9–10%). Therefore, we believe that further efforts will be devoted to this direction in the future^26,34^. In the present work, the yield strength on the order of 1.5 GPa along with ~16% elongation have rarely been achieved in existing HEAs (Fig. 5b). Hence our DPHL HEAs and some other ultrastrong HEAs^26,34^ with ultrahigh yield strength and good ductility expand known performance boundaries and exhibit great potential to improve energy efficiency and system performance in numerous fields, such as aerospace, transportation, and civilian infrastructure. More importantly, it demonstrates that inheriting the composite lamellar nature from the as-cast EHEA can be an effective way to prepare high-performance HEAs.

In this work, we successfully achieve a superior strength–ductility combination in AlCoCrFeNi_2.1_ EHEAs by introducing two concurrent effects, the two-hierarchical constraint effect and the self-generated microcrack-arresting mechanisms. The underlying origin lying behind the observed mechanical response is the inherited lamellar geometry from the as-cast EHEA. It is known that grain refinement to the nano/ultrafine-grained regime can render material stronger, but this process is usually accompanied by a dramatic loss of ductility^1–5^. However, with a composite lamellar architecture, the ultrafine-grained duplex microstructure in this study achieves an improved strength–ductility synergy. Phase decomposition is applied to optimize properties instead of just being a phase-instability phenomenon frequently observed in single-phase HEAs, which may encourage further research in this area. In addition, the AlCoCrFeNi_2.1_ EHEA could be simply processed to achieve properties that are an improvement over traditional TRIP (transformation-induced plasticity) and TWIP (twinning-induced plasticity) steels (Fig. 5). We believe that the simplicity in the present processing procedure is extremely attractive for industrial applications. Meanwhile, we anticipate that the investigated EHEA based on the present strengthening strategy can be further modified to satisfy more demanding application requirements, such as high-temperature and/or corrosion environments^6,11,12,15^. In conclusion, our results provide a promising pathway to strengthen eutectic alloys and prepare high-performance HEAs.

## Methods

### Sample preparation

The ingots with a nominal composition of AlCoCrFeNi_2.1_ (at%) was prepared by arc-melting a mixture of the constituent elements (purity better than 99.9 wt%) in a Ti-gettered high-purity argon atmosphere. The melting was repeated at least five times to achieve a good chemical homogeneity of the alloy. The molten alloy was suction-cast into a 30 mm (width) × 100 mm (length) × 6 mm (thickness) copper mold. Small pieces [dimensions: 50 mm (length) × 25 mm (width) × 4 mm (thickness)] were extracted from the as-cast material and subjected to multi-pass cold-rolling to 84–86% reduction in thickness (the final thickness of ~600 μm) using a laboratory-scale two-high rolling machine. The cold-rolled sheets were non-isothermally annealed to various temperatures. More specifically, four samples were, respectively, annealed from room temperature at 660, 700, 740, and 900 °C with the constant heating rate of 10 °C min^−1^, held at these four temperatures for 1 h and then water quenched immediately. We denote them as DPHL660, DPHL700, DPHL740, and DPHL900, respectively, in this study. Note that by the extraction of small pieces from the as-cast material, we can obtain high-quality samples with no or few surface defects (such as oxide films), smooth surfaces, and uniform thickness, for the subsequent cold-rolling and annealing treatment, which is able to prevent other factors to influence and even deteriorate mechanical properties. Please see ref. ^24^, which shows the related equations and procedures for calculating the back-stress and effective stress from the loading–unloading–reloading (LUR) curve.

### Microstructure characterization and tensile test

The EBSD and SEM observations were conducted by the CamScan Apollo 300 SEM equipped with a HKL–Technology EBSD system. The TEM analyses were operated on JEM–2100 F at 200 kV. The samples for EBSD and TEM were prepared, using a mixture of the 90% ethanol and 10% perchloric acid (vol.%). All tensile specimens were dog-bone-shaped, with a gauge length of 15 mm, a width of 3.2 mm, and a thickness of 600 μm. In terms of the ultrafine-grained material, here, such tensile dimensions are typically consistent with that frequently reported in the literature^16,18^. Meanwhile, there are over 400 grains along the thickness direction in specimens (Fig. 1 and Supplementary Fig. 1a, b), which far exceeds the grain number (~100) required theoretically to ensure the representativeness and reproducibility of tensile data at a larger scale. Moreover, to obtain the reproducible tensile property, all tensile tests were repeated five times. The direction of tensile tests was parallel to the rolling direction. Tensile tests were carried out at room temperature using an MTS Criterion Model 44 with an initial strain rate of 2.5 × 10^−4^ s^−1^. The condition for LUR tests was the same as that of the monotonic tensile test. Upon straining to a designated strain at the strain rate of 2.5 × 10^−4^ s^−1^, the specimen was unloaded by the stress-control mode to 20 N at the unloading rate of 200 N min^−1^, followed by reloading at a strain rate of 2.5 × 10^−4^ s^−1^ to the same applied stress before the next unloading. All tensile tests were conducted, using a 12-mm extensometer to monitor the strain.

## Supplementary information

Supplementary Information

## Data Availability

The data that support the findings of this study are available from the corresponding authors upon reasonable request.

## References

[1] Wang YM, Chen MW, Zhou FH, Ma E (2002). High tensile ductility in a nanostructured metal. Nature.

[2] Wang YM (2018). Additively manufactured hierarchical stainless steels with high strength and ductility. Nat. Mater..

[3] Lu K (2014). Making strong nanomaterials ductile with gradients. Science.

[4] Huang HL (2017). Phase-transformation ductilization of brittle high-entropy alloys via metastability engineering. Adv. Mater..

[5] Ma E, Zhu T (2017). Towards strength–ductility synergy through the design of heterogeneous nanostructures in metals. Mater. Today.

[6] Lu YP (2014). A promising new class of high-temperature alloys: eutectic high-entropy alloys. Sci. Rep..

[7] Wani IS (2016). Ultrafine-grained AlCoCrFeNi_2.1_ eutectic high-entropy alloy. Mater. Res. Lett..

[8] Tan YZ (2012). Microstructures, strengthening mechanisms and fracture behavior of Cu–Ag alloys processed by high-pressure torsion. Acta Mater..

[9] Jana S, Mishra RS, Baumann JB, Grant G (2010). Effect of friction stir processing on fatigue behavior of an investment cast Al–7Si–0.6 Mg alloy. Acta Mater..

[10] Wang L, Shen J, Shang Z, Fu HZ (2014). Microstructure evolution and enhancement of fracture toughness of NiAl–Cr(Mo)–(Hf,Dy) alloy with a small addition of Fe during heat treatment. Scr. Mater..

[11] Guo S, Ng C, Liu CT (2013). Sunflower-like solidification microstructure in a near-eutectic high-entropy Alloy. Mater. Res. Lett..

[12] Lu YP (2017). Directly cast bulk eutectic and near-eutectic high entropy alloys with balanced strength and ductility in a wide temperature range. Acta Mater..

[13] Jin X, Zhou Y, Zhang L, Du XY, Li BS (2018). A novel Fe_20_Co_20_Ni_41_Al_19_ eutectic high entropy alloy with excellent tensile properties. Mater. Lett..

[14] Yeh JW (2004). Nanostructured high-entropy alloys with multiple principal elements: novel alloy design concepts and outcomes. Adv. Eng. Mater..

[15] Ye YF, Wang Q, Lu J, Liu CT, Yang Y (2016). High-entropy alloy: challenges and prospects. Mater. Today.

[16] Wu XL (2015). Heterogeneous lamella structure unites ultrafine-grain strength with coarse-grain ductility. Proc. Natl Acad. Sci. USA.

[17] Wu XL, Zhu YT (2017). Heterogeneous materials: a new class of materials with unprecedented mechanical properties. Mater. Res. Lett..

[18] Yang MX (2016). Strain hardening in Fe–16Mn–10Al–0.86C–5Ni high specific strength steel. Acta Mater..

[19] Schuh B (2015). Mechanical properties, microstructure and thermal stability of a nanocrystalline CoCrFeMnNi high-entropy alloy after severe plastic deformation. Acta Mater..

[20] Otto F (2016). Decomposition of the single-phase high-entropy alloy CrMnFeCoNi after prolonged anneals at intermediate temperatures. Acta Mater..

[21] He F (2017). Phase separation of metastable CoCrFeNi high entropy alloy at intermediate temperatures. Scr. Mater..

[22] Gwalani B (2018). Modifying transformation pathways in high entropy alloys or complex concentrated alloys via thermo-mechanical processing. Acta Mater..

[23] Bhattacharjee T (2018). Simultaneous strength–ductility enhancement of a nano-lamellar AlCoCrFeNi_2.1_ eutectic high entropy alloy by cryo-rolling and annealing. Sci. Rep..

[24] Yang MX, Pan Y, Yuan FP, Zhu YT, Wu XL (2016). Back stress strengthening and strain hardening in gradient structure. Mater. Res. Lett..

[25] Qi L, Chrzan DC (2014). Tuning ideal tensile strengths and intrinsic ductility of bcc refractory alloys. Phys. Rev. Lett..

[26] Yang MX (2018). Dynamically reinforced heterogeneous grain structure prolongs ductility in a medium-entropy alloy with gigapascal yield strength. Proc. Natl Acad. Sci. USA.

[27] Li Y, Li W, Min N, Liu WQ, Jin XJ (2017). Effects of hot/cold deformation on the microstructures and mechanical properties of ultra-low carbon medium manganese quenching-partitioning-tempering steels. Acta Mater..

[28] Huang LJ, Geng L, Peng HX (2015). Microstructurally inhomogeneous composites: is a homogeneous reinforcement distribution optimal?. Prog. Mater. Sci..

[29] Liu ZY, Xiao BL, Wang WG, Ma ZY (2013). Developing high-performance aluminum matrix composites with directionally aligned carbon nanotubes by combining friction stir processing and subsequent rolling. Carbon N. Y..

[30] Wu H (2017). Deformation behavior of brittle/ductile multilayered composites under interface constraint effect. Int. J. Plast..

[31] Wang ZW, Baker I, Guo W, Poplawsky JD (2017). The effect of carbon on the microstructures, mechanical properties, and deformation mechanisms of thermo-mechanically treated Fe_40.4_Ni_11.3_Mn_34.8_Al_7.5_Cr_6_ high entropy alloys. Acta Mater..

[32] He JY (2016). A precipitation-hardened high-entropy alloy with outstanding tensile properties. Acta Mater..

[33] Baker I, Meng FL, Wu M, Brandenberg A (2016). Recrystalliztion of a novel two-phase FeNiMnAlCr high entropy alloy. J. Alloy Compd..

[34] Liang YJ (2018). High-content ductile coherent nanoprecipitates achieve ultrastrong high-entropy alloys. Nat. Commun..

[35] Li ZM, Pradeep KG, Deng Y, Raabe D, Tasan CC (2016). Metastable high-entropy dual-phase alloys overcome the strength–ductility trade-off. Nature.

