# Supplementary material for: Enhanced strength–ductility synergy in ultrafine-grained eutectic high-entropy alloys by inheriting microstructural lamellae
Source: Nat Commun. 2019 Jan 30;10:489. doi: 10.1038/s41467-019-08460-2 (PMC6353877; doi:10.1038/s41467-019-08460-2)
Supplement: Supplementary file 1 — Supplementary Information [file 41467_2019_8460_MOESM1_ESM.pdf]

Supplementary Information

Enhanced strength–ductility synergy in ultrafine-grained eutectic high-entropy alloys by inheriting microstructural lamellae

Shi et al.

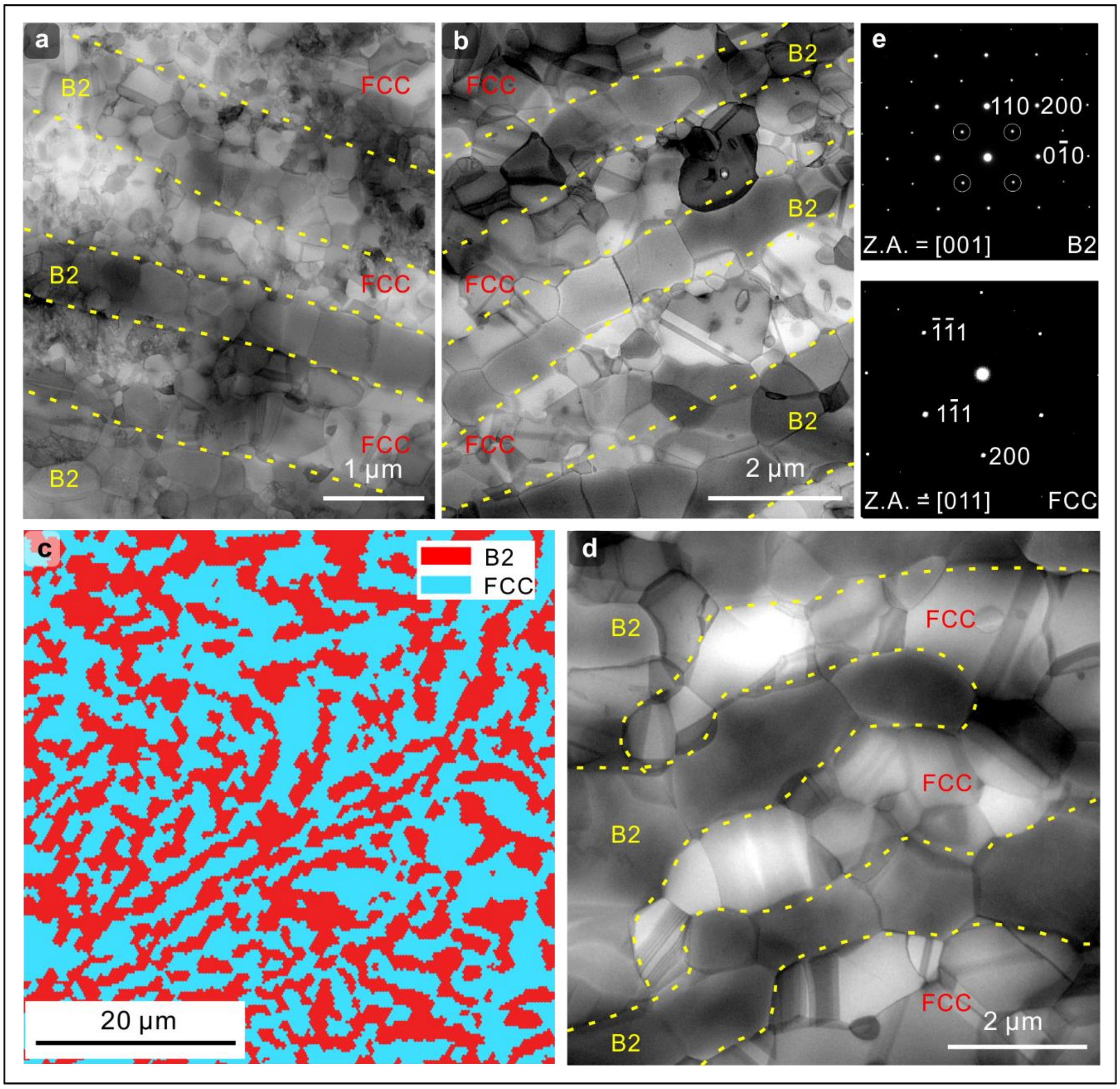

Supplementary Figure 1 | Microstructures of the hierarchical DPHL HEAs. STEM images of (**a**) DPHL660 and (**b**) DPHL740 showing the DPHL structure. The alternate FCC and B2 ultrafine-grained lamellae are marked by dashed yellow lines. Due to the slightly-low annealing temperature in DPHL660, there are a few regions in which grains with an apparently-high dislocation density. But the proportion of such an area is not exceeding 15%, which is also one of the evaluation criteria for an optimal DPHL structure in this paper. (**c**) EBSD-phase image and (**d**) STEM image of D-DPHL900 (non-isothermally annealed at 900 ℃) exhibiting a degraded lamella structure (indicated by dashed yellow lines) owning to the excessively-high annealing temperature, thus inferior tensile properties (Supplementary Fig. 6). (**e**) The related SADPs of DPHL700. Superlattice-diffraction spots are indicated by circles.

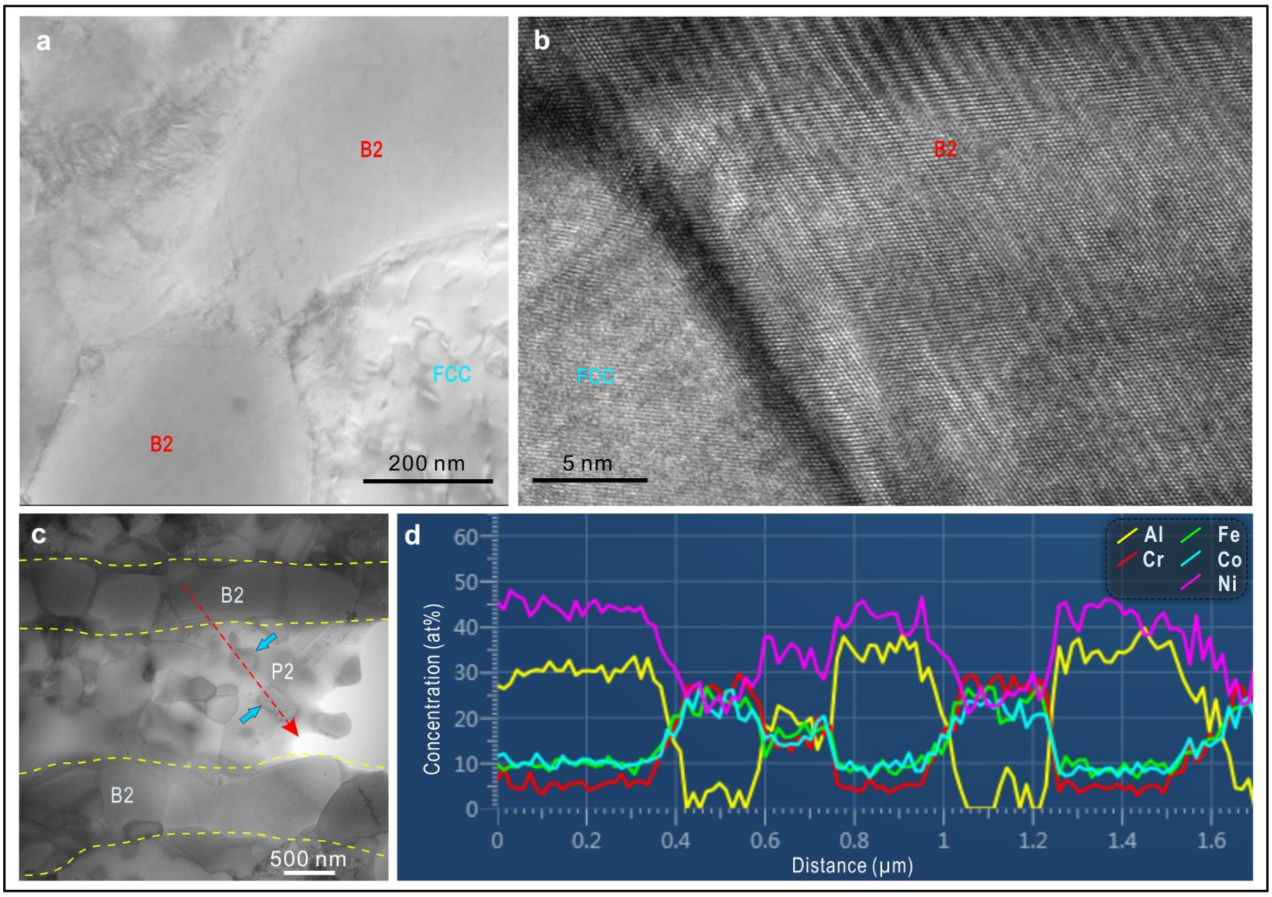

Supplementary Figure 2 | Microstructure and composition profile of the hierarchical DPHL HEA. (**a**) STEM image of the DPHL sample after the tensile strain of ~6%, which does not show any nano-precipitates, and the interaction between precipitates and dislocations in the B2 phase. (**b**) The corresponding high-resolution TEM image of the B2 phase, which isn't decorated with any nano-precipitates. (**c**) STEM image of the DPHL structure. Here, the intergranular B2 phase (P2) marked by blue arrows, and the B2 lamellae indicated by yellow dashed lines. The red dashed arrow indicates the direction of the EDS-linear scanning. (**d**) The corresponding EDS composition profile of the selected region marked by a red dashed arrow in (**c**). Obviously, both of the intergranular B2 phase and the B2 lamellae are composed of dominant Ni and Al with minor other three elements. Here, it is closer to 1:1 in the ratio of Ni and Al elements for the intergranular B2 phase.

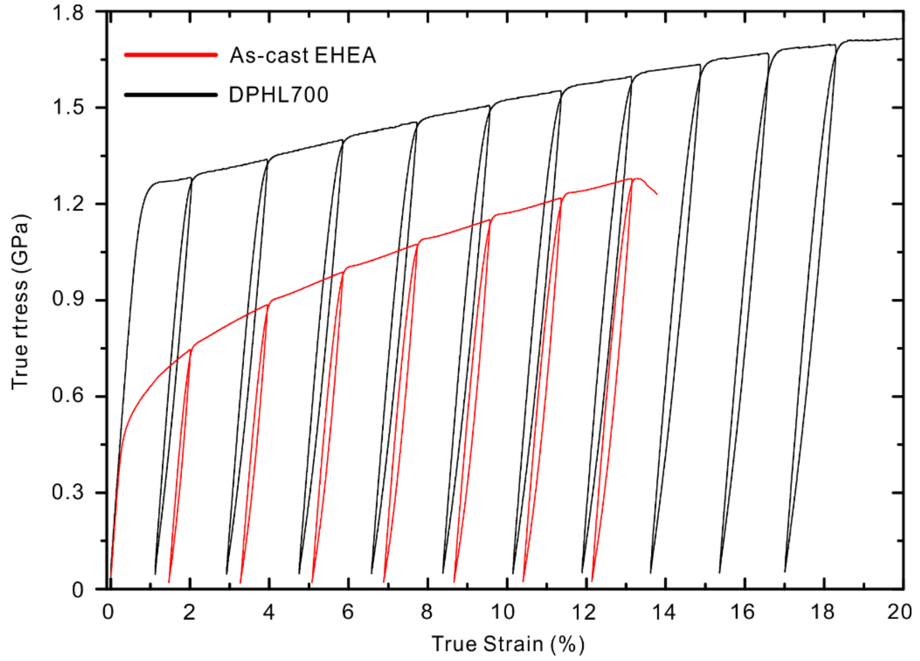

Supplementary Figure 3 | The whole loading–unloading–reloading true stress–strain curves for the hierarchical DPHL700 and the as-cast EHEA.

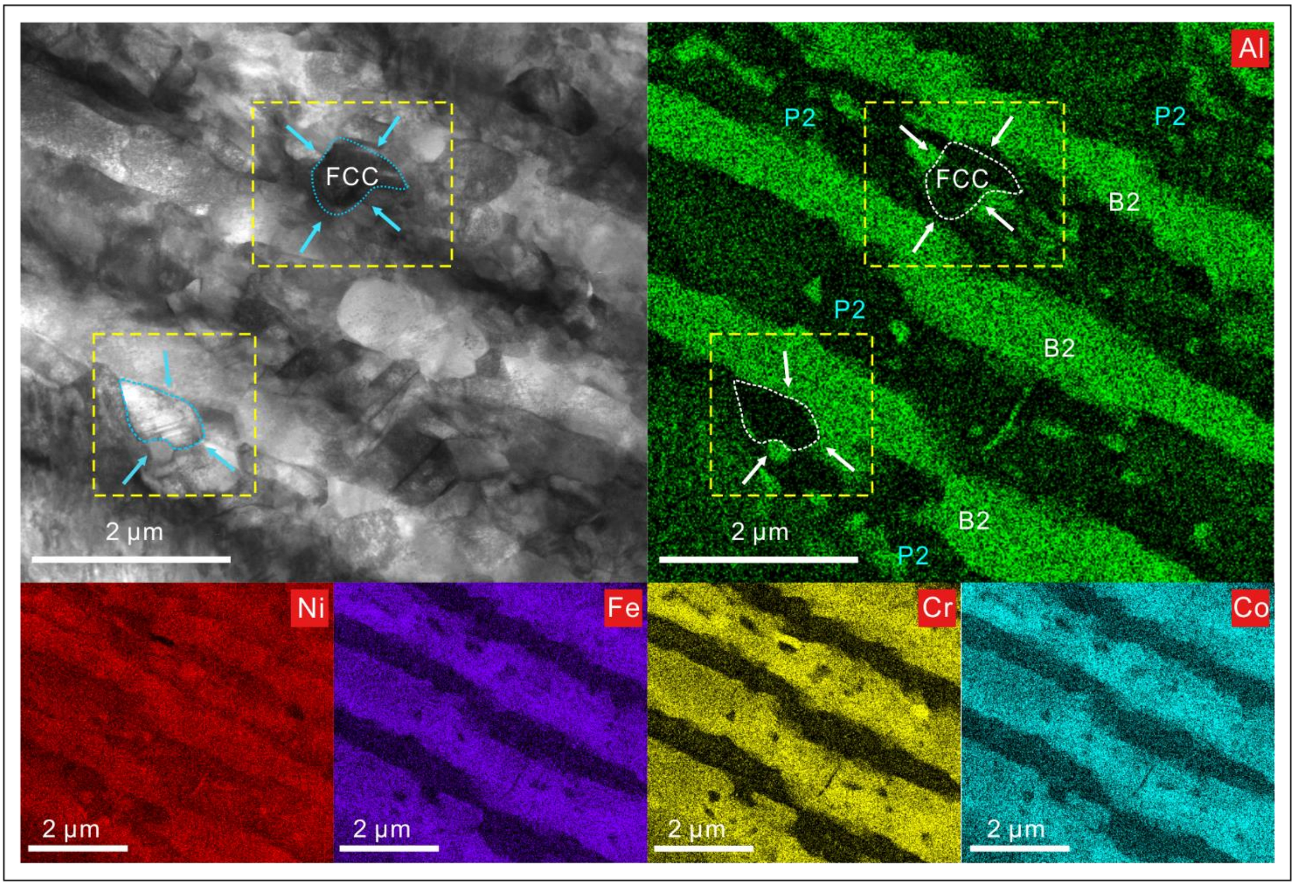

Supplementary Figure 4 | Microstructure and the related EDS maps of the hierarchical DPHL HEA stretched to fracture. STEM image and related EDS maps displaying the obviously-inhomogeneous plastic deformation and irregular lamella interfaces. The compositional distributions in P2 (the intergranular B2 grains) and dual-phase lamellae are the same as the elemental partitioning shown in Fig. 1e. The hard P2 can also plastically deform, evidenced by the aspect-ratio change[16,17] from roughly-equiaxed grains (Fig. 1e) to present extended ones. In the yellow frames, there show that the FCC grains deform along various directions, due to the two-level constraint effect from the micron-size B2 lamellae and the submicron-size P2 (marked by blue and white arrows).

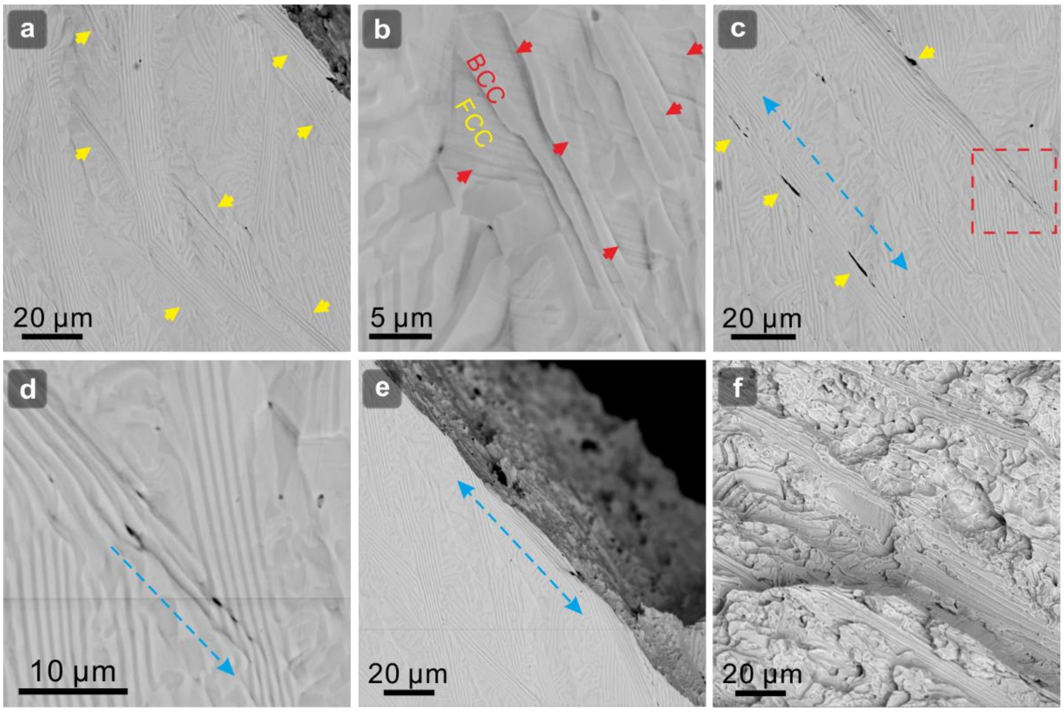

Supplementary Figure 5 | Typical SEM images of damage-evolution mechanisms for the as-cast EHEA. (**a**) Some lamellar microdomains with obvious strain localization (indicated by yellow arrows) near the fractured end. (**b**) High-magnification SEM image exhibiting the stress concentration (local slip bands, highlighted by red arrows) in FCC lamellae near phase interfaces. (**c**) The microcracks (indicated by yellow arrows) nucleated primarily at the phase interfaces, due to the high strain localization. The blue double-headed arrow shows the crack-propagation direction. (**d**) Enlarged SEM image in (**c**) showing the microcracks nucleated at the intersection of two lamellar domains with distinct lamellar directions. The blue arrow indicates the crack-propagation direction. (**e**) The cracks nucleated in these lamellar microdomains with the high strain concentration, as a fuse (indicated by a double-headed arrow), triggering the whole fracture. (**f**) Fractography of the fracture surface mainly featuring a trench-like microstructure, which reveals a brittle and ductile mixed fracture mode.

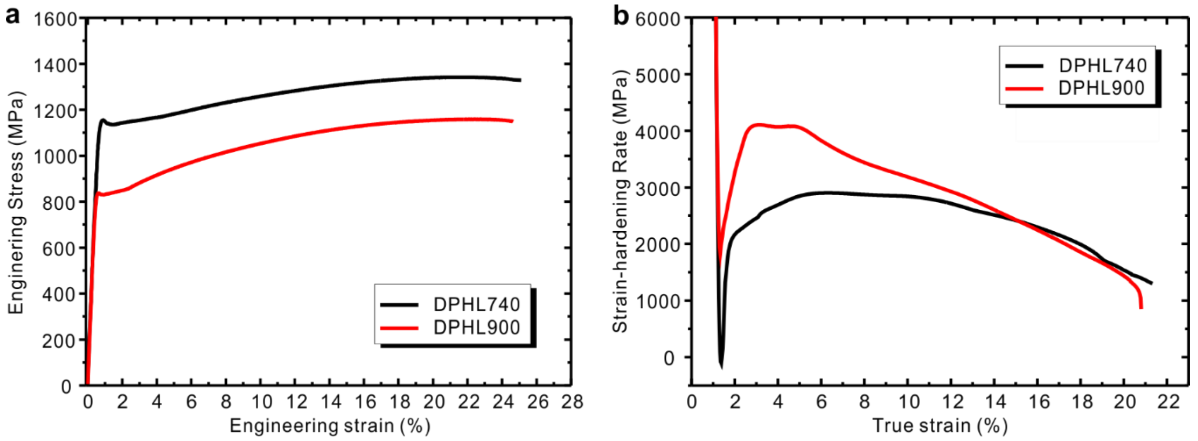

Supplementary Figure 6 | Mechanical responses of DPHL740 and D-DPHL900 at room temperature. (**a**) Tensile properties. The DPHL740 and D-DPHL900 prepared by the non-isothermally annealing treatment from the room temperature to 740 ℃ and 900 ℃, respectively, with a constant heating rate of 10 ℃ min^{-1}, held at this temperature for 1h and then water quenched immediately. Due to the less ideal, partially-degraded DPHL structure (Supplementary Fig. 1c, d), the D-DPHL900 exhibits an obviously lower yield strength but without a corresponding improvement in ductility, in comparison to the DPHL740. (**b**) Strain-hardening response. The ability of sustaining the high strain-hardening rate in D-DPHL900 is significantly lower than that in DPHL740. After the strain of ~5%, the work-hardening rate is continuously decreasing and even lower than that of DPHL740 (at the strain of ~15%).

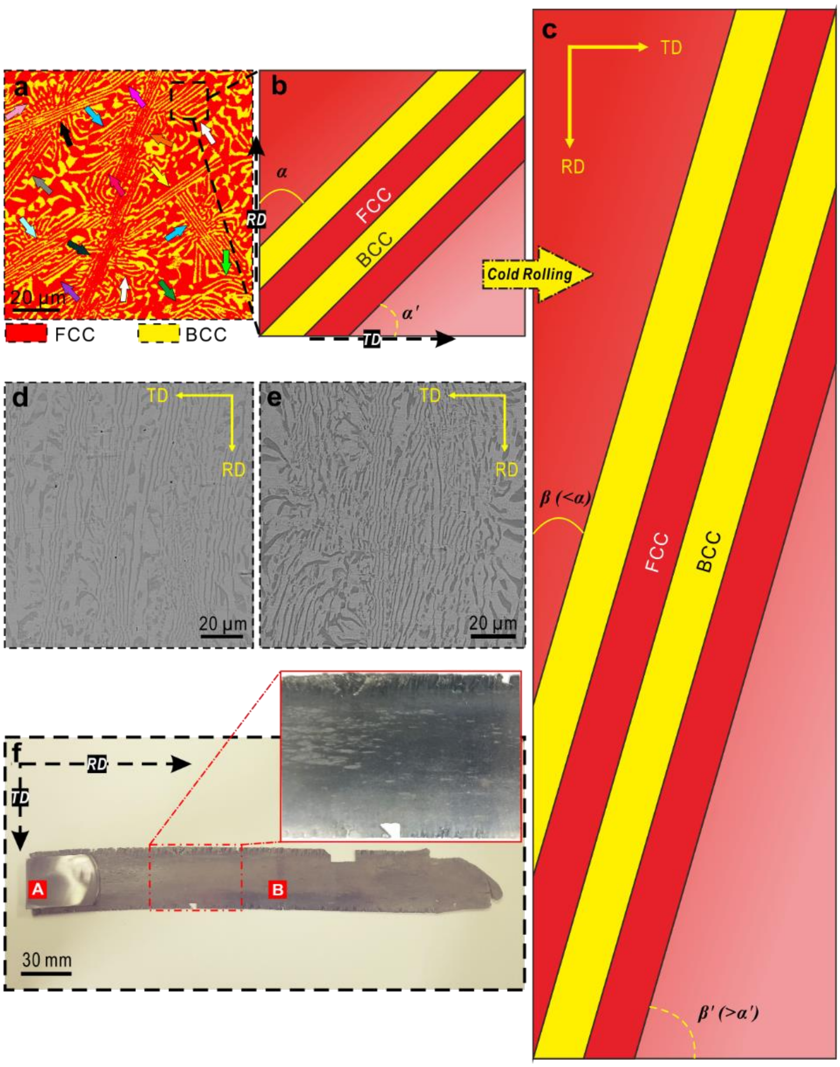

Supplementary Figure 7 | (**a**) EBSD-phase image of the as-cast EHEA showing a random lamellar alignment direction, such as those lamellae marked by arrows with different colors, among different eutectic colonies. (**b**) A lamellar alignment diagram of the selected eutectic colony marked by the black dashed square in (**a**). Here, the black dashed arrow between (**a**) and (**b**) indicates the rolling direction (RD). (**c**) A corresponding lamellar alignment diagram after the rolling deformation for (**b**). Due to the great degree of elongation along RD after rolling [as shown in (**f**)], the intersection angle β between the lamellar alignment direction and the RD is much smaller than that α in (**b**), thus exhibiting an aligned lamellar arrangement along RD. In this study, if the initial α = 45 °(**b**), the resulting β = 10–14 °(**c**) in theory. (**d**) SEM image of the rolled EHEA exhibiting massive special lamellae (~82–87 vol.%) with a directionally-aligned arrangement along RD. In fact, processing the aligned arrangement via the rolling deformation has been extensively applied in developing the directionally-aligned carbon nanotubes for high-performance metal matrix composites[28]. (**e**) SEM image of the DPHL EHEA. Surprisingly, these aligned lamellae still exist even after the non-isothermally annealing treatment (700 ℃) for the rolled EHEA in (**d**). Additionally, some other features are also retained, such as the varied inter-lamella spacings and the existence of some short, thick lamellae. In other words, we can't tell, in

some degree, the microstructure distinction between the cold-rolled EHEA (**d**) and the DPHL EHEA (**e**) only from SEM images. As discussed above, thus, we regard the aligned arrangement behavior as that inheriting the lamellar nature from the as-cast EHEA. (**f**) Surface morphology of the as-cast sample (A) and the corresponding cold-rolled ~85% sample (B). As shown in the inset, there are no cracks on the rolled specimen surface, and only small and short cracks distributed on both side of the rolled sheet. (RD, rolling direction. TD, transverse direction.)

Supplementary Table 1 | The tensile properties and the yield ratios of the hierarchical DPHL HEAs, as-cast EHEA, and ultrafine-grained (UFG) EHEA

Material	Yield strength (MPa)	Tensile strength (MPa)	Ductility (%)	Yield ratio (%)	Comment
As-cast EHEA	512 ±26	1,080 ±23	16.2 ±0.8	47.41	This work
UFG EHEA	~1,100	~1,200	~12.0	91.67	Ref. 7
DPHL740	1,154 ±9	1,340 ±15	25.0 ±1.1	86.12	This work
DPHL700	1,263 ±16	1,442 ±19	21.4 ±0.9	87.59	This work
DPHL660	1,490 ±17	1,638 ±25	16.0 ±0.5	90.96	This work

The tensile property of the as-cast EHEA in the present work is almost the same with that in ref. 12.

The use of "~" rather than "±" is due to the fuzzy range of the mechanical property exhibited in ref. 7.
